# Fluorine-free electrolytes for all-solid sodium-ion batteries based on percyano-substituted organic salts

**DOI:** 10.1038/srep40036

**Published:** 2017-01-09

**Authors:** Anna Bitner-Michalska, Gene M. Nolis, Grażyna Żukowska, Aldona Zalewska, Marcin Poterała, Tomasz Trzeciak, Maciej Dranka, Michał Kalita, Piotr Jankowski, Leszek Niedzicki, Janusz Zachara, Marek Marcinek, Władysław Wieczorek

**Affiliations:** 1 Polymer Ionics Research Group, Faculty of Chemistry Warsaw University, of Technology, Noakowskiego 3, 00-664 Warsaw, Poland.

## Abstract

A new family of fluorine-free solid-polymer electrolytes, for use in sodium-ion battery applications, is presented. Three novel sodium salts withdiffuse negative charges: sodium pentacyanopropenide (NaPCPI), sodium 2,3,4,5-tetracyanopirolate (NaTCP) and sodium 2,4,5-tricyanoimidazolate (NaTIM) were designed andtested in a poly(ethylene oxide) (PEO) matrix as polymer electrolytes for anall-solid sodium-ion battery. Due to unique, non-covalent structural configurations of anions, improved ionic conductivities were observed. As an example, “liquid-like” high conductivities (>1 mS cm^−1^) were obtained above 70 °C for solid-polymer electrolyte with a PEO to NaTCP molar ratio of 16:1. All presented salts showed high thermal stability and suitable windows of electrochemical stability between 3 and 5 V. These new anions open a new class of compounds with non-covalent structure for electrolytes system applications.

Interest in sodium-ion batteries has increased in order to supplement the market held by lithium-ion cells^1^. The emergence of sodium as a contender to lithium stems from its high abundance, low-cost, suitable redox potential (*E*˚(Na^+^/Na) = −2.71 V, not much less in absolute value than *E*˚(Li^+^/Li) = −3.05 V *vs.* standard hydrogen electrode) and ability to intercalate into both positive and negative electrode materials^2^.

Research has rapidly identified viable cathodes and anodes able for reversible sodium intercalation. And yet, it is generally accepted that the electrolyte limits the usage of new high-voltage cathode materials or rapid kinetics of cation intercalation. Not many studies on electrolytes have been reported for Na-ion cells, but the ones that exist focus on conventional, liquid-based systems^3^.

There has been considerably less research focused on solid-polymer electrolytes due to their poorer ionic conductivity at room temperature and reduced interfacial surface area between the electrolyte and electrode. Nonetheless, continued interest in such technologies stems from their processing ability and flexibility, higher safety due to the absence of flammable organic solvents and high dimensional stability^4^,^5^,^6^,^7^,^8^,^9^,^10^,^11^,^12^,^13^. Despite not being fluid in a wide temperature range, polyethers were first shown to dissolve inorganic salts and conduct resulting ions at room temperature in the 1970s^14^. Of these, poly(ethylene oxide) (PEO) took center stage of lithium electrolyte research in the 1990s.

Reported salts for PEO-based, solid-polymer electrolytes in sodium-ion battery applications include sodium trifluoromethane sulfonate (NaCF_3_SO_3_)^15^,^16^,^17^,^18^, sodium thiocyanate (NaSCN), sodium tetraflouroborate (NaBF_4_)^19^, sodiumbis(trisfluoromethanesulfonyl)imide (NaTFSI)^20^,^21^,^22^, sodium perchlorate (NaClO_4_)^23^ and sodium hexafluorophosphate (NaPF_6_)^24^. Generally, these membranes and their lithium analogues show ionic conductivities on the order of 10^−5^ S cm^−1^ or less at room temperature, which is not enough for rechargeable batteries^25^,^26^. NaTFSI is a promising salt as it shows higher ionic conductivities in PEO-based, solid-polymer electrolytes at room temperature (0.1 mS cm^−1^) and about 1 mS cm^−1^ above 80 °C^21^.

While the best conductivities have been shown by fluorinated salts so far, there is a paradigm shift in research and development. Since fluorinated salts may negatively affect electrolyte safety, cost and environmental friendliness, alternative fluorine-free options have been investigated^27^. Even partial elimination of the salts containing fluorine (both type YF_X_^n−^ or fluoro-organic) should lead to a reduction in production and emission of toxic compounds to the environment. Forgoing the use of a salt, which doesn’t contain fluorine, may also increase the stability of the electrode-electrolyte phase boundary in batteries or simply not contribute to the formation of blocking lithium fluoride layer at the solid-electrolyte interphase.

Jónnson and Johannson recently argued, using DFT calculations, that anions with a diffuse negative charge will have lower cation-anion interaction energies^28^. Following, it is expected that these salts dissociate more in electrolytes, leading to an increase in the number of effective charge carriers. In particular, aromatic (Hückel-type) percyano-anions were calculated to have lower interaction energies with Na^+^ than Li^+^ cations. Such anions are characterized by conjugated π-system covering an entire anion including the terminal cyano groups.

The Polymer Ionics Research Group has previously shown that the electron-withdrawing cyano substituents, incorporated in highly delocalized electron system, reduce the basicity of the anion; inhibiting electron donation toward alkali metal cations^29^. Moreover, introducing additional cyano substituents decreases the basicity of parent anion even more. For example, thep*K*_b_ of sodium 2,3,4,5-tetracyanopirolateanion is quite high, 11.29, but is expected to have low interaction with Na^+ ^30.

In this study, sodium pentacyanopropenide (NaPCPI), sodium 2,3,4,5-tetracyanopirolate (NaTCP)and sodium 2,4,5-tricyanoimidazolate (NaTIM) (Fig. 1) were dissolved in liquid and solid-polymer electrolytes. These salts were chosen based on their fluorine-free composition and anions with diffuse negative charges. Their performance as electrolytes were benchmarked against NaPF_6_. In order to assess their viability in sodium-ion batteries, their thermal stability, salt-solvent interactions, electrochemical stability and ionic conductivities were characterized.

## Results

### Structure of crystalline sodium solvates with percyano anions

**Figure 2:**
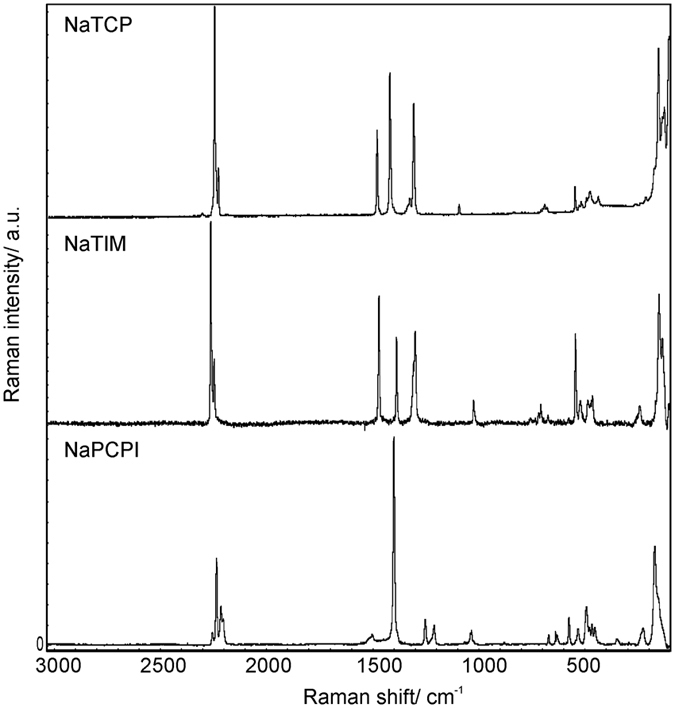
Raman spectra of pure salts.

Novel fluorine-free salts (NaPCPI, NaTCP and NaTIM) were synthesized according to the procedures detailed in the methods section. Raman and NMR spectroscopy experiments were used to confirm the structure and purity of the as-synthesized salts. Characteristic chemical shifts of each salts’ NMR spectra are reported in the methods chapter. The Raman spectrum of NaPCPI shows multiple nitrile ν(CN) stretching bands near 2250–2180 cm^−1^; resulting from its linear structure^31^. The strong band at 1400 cm^−1^ is related to ν_C-C-C_ vibration; wherein this linear backbone a delocalized electron is shared between the carbon atoms via π bonding (Fig. 2.).

Raman spectrum of pure NaTCP possesses nitrile ν_CN_ vibrations near 2245 and 2230 cm^−1^, ring stretching vibrations between 1500–1300 cm^−1^ and C = C in-plane bending vibrations below 1200 cm^−1^. These vibrations are characteristic for an anion composed of a percyano-substituted pyrrole ring. The final salt’s, NaTIM, Raman spectrum contains multiple excitation bands in the region of 1500–1250 cm^−1^ from ring ν_C=C_ and ν_C=N_ vibrations and nitrile ν_CN_ vibrations at 2260 and 2250 cm^−1^. These results indicate that the anion (TIM^−^) is composed of a percyano-subtituted imidazole ring. Both TCP^−^ and TIM^−^ anions fulfill Hückel’s rule, possessing planar ring geometries, and contain a delocalized negative charge through conjugated π bonds.

In order to confirm the molecular assignments in that spectra, solvates of the salts with 12-Crown-4 ether(12C4) have been prepared, with subsequent x-ray crystal structure determination performed on single crystals. A summary of solvate single crystal diffraction pattern refinements are shown in Supporting Table 1. Notably, single crystal solvates of Na(12C4)_2_^+^ PCPI^−^ and Na(12C4)_2_^+^ TCP^−^ have triclinic symmetry (space group *P1*) and Na(12C4)_2_^+^ TIM^−^ possesses monoclinic symmetry (space group *P2*_*1*_*/n*). Supporting Figures 1–3. show refined molecular structures of the aforementioned solvates, respectively. In general, these figures show the solvates comprised of a sodium cation trapped between two crown ether rings. This spatial configuration isolates the anion, providing a model system for a “free” anion. The molecular structure of anions, in the three new solvates, were revealed to be of planar geometries. This further suggests delocalization of the negative charge through π bonds over the entire anions.

On a closer inspection of packing arrangement in crystal structure of Na(12C4)_2_^+^ TCP^−^, it is discovered that there are a noteworthy π-stacking interactions between TCP^−^ anions (Supporting Figure 4). Supporting Figure 5, shows columnar stacking of anions forming tetrameric units. Its occurrence was also confirmed by calculations (Supporting Figure 6). The simulations confirm the energetically favorable stacking of TCP^−^ anions with an energy minimum between 3.0 and 3.5 Å (Supporting Table 2).

Presence of this type of anionic aggregation indicates that π-π interactions between percyanoHückel-type anions, driven by dispersion forces, are supported by weak electrostatic interactions to counter ions. This ability of anions to form tetrameric columns due to intermolecular π-π interactions, which has not been considered before in aspects of electrolyte structure, and seems to profoundly alter the properties of electrolytes. Recently, Becker *et al*. showed that TCP^−^ anionsinteract with weakly coordinating cations such as Me_4_N^+^ or EMIM^+^ to form isolated ion pairs, in which anion-anion π-π stacking is present^32^. Observing such a unique coordinating character between anions of NaTCP suggests that these types of noncovalent interactions are crucial for salt aggregation in polymer matrix.

**Figure 3:**
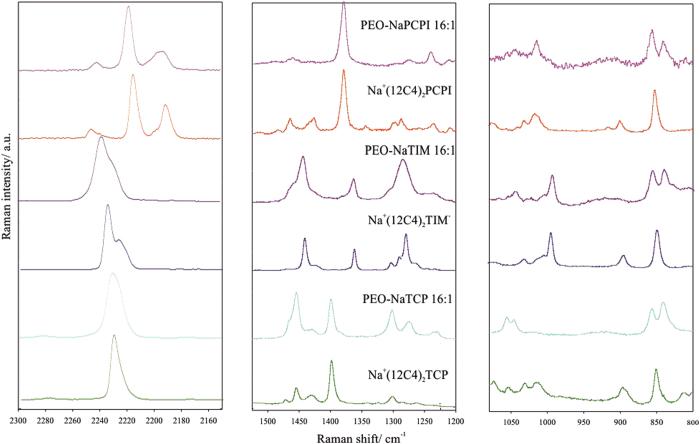
Raman spectra of PEO-Na-salts complexes.

Raman spectra of Na-salts dissolved in a PEO matrix are shown in Supporting Figures 7–10 with PEO:Na molar ratios of 10:1 and 16:1. PEO:Na concentrations of 16:1 are expected to have dissociated salts, unlike membranes of 10:1, and should not have significant amounts of ion pairing. Therefore, these systems also serve as a convenient model of free PCPI^−^, TCP^−^ and TIM^−^ anions dissolved in a solid-polymer matrix. Figure 3 shows the Raman spectra of PEO-based membranes with novel salts (16:1) alongside their crown ether complex analogues to compare the structure of the “free” anions.

In regards to solid-polymer electrolytes, most of the salt is expected to exist in the form of free ions, accompanied with relatively small amounts of ionic pairing. In fact, vibrational band positions, selected as probes of ionic association, are very close to that observed in spectra of model solvate systems, as shown in Fig. 3. The bands due to ν_CH2_ and rocking vibrations deliver information concerning conformations of the polyether chain. This, in turn, depends on the type of crystalline and amorphous phases, such as coordination of the polymer with a cation. At an O:Na molar ratio of 16:1, the spectral patterns of the studied membranes are close to that of pure PEO. Slightly different behavior was exhibited by membranes doped with higher amounts of the salt (O:Na equal to 10:1), where the bands typical for the crystalline PEO phase are overlapped with bands of an amorphous phase of the complex (Supporting Figure 8)^15^,^19^,^23^. Recording of good quality Raman spectra for NaPCPI systems was not possible due to the strong fluorescence.

Similar tendencies were observed in FTIR spectra of the NaPCPI-based electrolytes (Supporting Figure 11). In the O:Na range spanning from 16 to 50 the spectral features of PEO membrane closely resemble that of pure PEO. An increase in the salt content results in decrease in the crystallinity of the samples, which can be concluded on the basis of gradual decrease in the intensity of the bands at 1360 and 1341 cm^−1^, typical for crystalline PEO. The formation of the polymer-salt complex and increasing of the salt aggregation at higher salt content is supported by increase in the intensities of bands peaking at 1085 and 2194 cm^−1^, and attributed to ν_c-o-c_and ν_CN_, attribmuted to PEO-NaPCPI complex.

### Electrochemical behavior of polymer electrolytes

**Figure 4:**
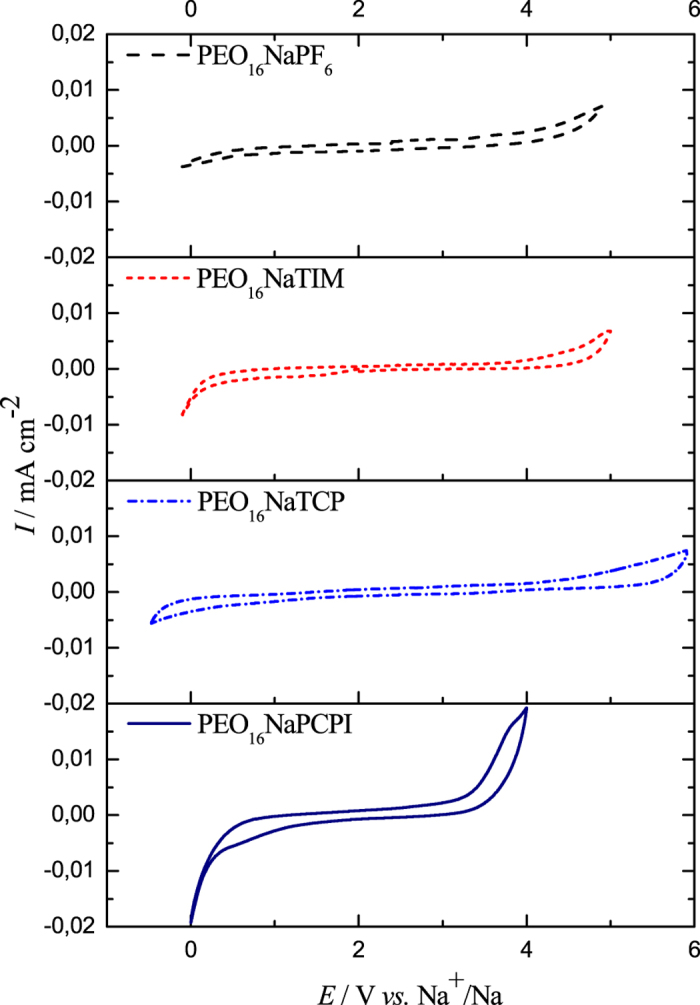
Cyclic voltammetry of electrolytes of PEO-based, solid-polymer electrolytes with salts of NaPF6, NaTIM, NaTCP and NaPCPI at 50 °C.

The electrochemical stability of electrolytes is a key parameter in terms of its application in sodium-ion batteries. Stability of each salt in PEO and PEG-500 (as a model of fully amorphous system) was determined. According to Fig. 4, the least stable salt was NaPCPI, with a stability window of about 3 V *vs.* Na^+^/Na. Even less stability this salt present in PEG-500, due to decomposition around 0 V. A stability window of approximately 5 V *vs.* Na^+^/Na was obtained for electrolytes with NaTCP and NaTIM. It easy to observed that polymer electrolyte containing NaTIM exhibited almost the same range of stability as those with NaPF_6_, around 0.7 V improvement we can observe for electrolyte containing NaTCP (also present the best conductivity).This trend is in agreement with predicted oxidation potentials (Supporting Table 3). The electrolyte containing NaPF_6_ did not decompose inside a window of 4.5 V *vs.* Na^+^/Na. Additionally, it has been shown by Moreno *et al*. that oxidation of PEO_20_NaTFSI membranes occurs above 4.8 V *vs*. Na^+^/Na^21^. Moreover, electrolytes based on PEG-500 present similar trends of stability window (Supporting Figure 12).

**Figure 5:**
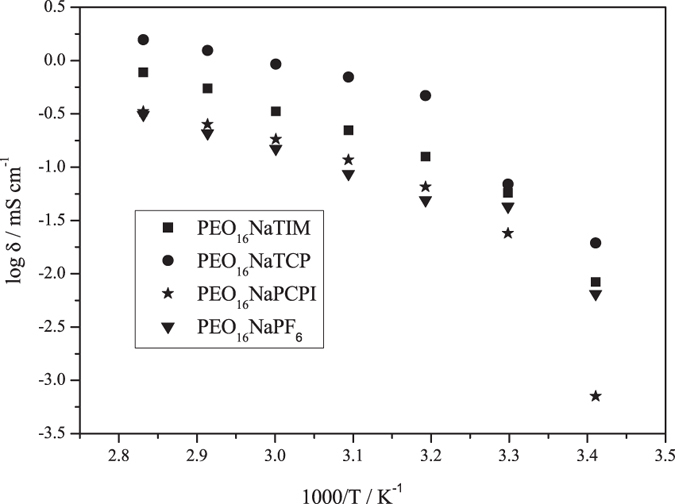
Ionic conductivity of solid-polymer electrolytes.

In order to determine ionic conductivity for solid-polymer and liquid electrolytes, electrochemical impedance spectroscopy experiments were performed on NaPF_6_, NaPCPI, NaTCP and NaTIM salts dissolved in PEO and PEG-500. Figure 5 shows an Arrhenius plot of the log of ionic conductivity (*σ*) versus 1000/T for PEO-based electrolytes; whereas Table 1 and Supporting Table 4 list measured *σ*for PEO- and PEG-based electrolytes, respectively. Solid-polymer electrolytes doped with NaPF_6_, NaTCP and NaTIM, at O:Na molar ratios of 16:1, showed optimal*σ* values to other concentrations; whereas, the optimum concentration for NaPCPI membrane was found to be 20:1. Ionic conductivity values of PEO-based electrolytes studied in this paper are comparable, if not better, to other Na-salts reported in the literature^20^,^21^,^24^.

PEO_16_NaPF_6_ exhibited *σ* of the order of 0.1 mS cm^−1^ above 60 °C. On the other hand, novel PCPI^−^ and Hückel-type anions showed ionic conductivities on the order of 0.1 mS cm^−1^ above 50 and 40 °C, respectively. At low temperatures (20 and 30 °C), PEO_20_NaPCPI exhibited the lowest ionic conductivity with *σ* = 0.0014 and 0.011 mS cm^−1^, respectively. However, Hückel-type anions present improved ionic conductivity values 0.069 and 0.057 mS cm^−1^ at 30 °C. At high temperature (70 °C), PEO_16_NaTCP showed ionic conductivity values (so-called “liquid-like”) greater than 1 mS cm^−1^. Other research groups report such conductivities at or above 80 °C for PEO_20_NaTFSI membranes, however this salt contains fluorine^20^,^21^. Additionally, and according to Supporting Table 4, electrolytes based on PEG-500 present similar trends.

From these results, it is clear that ionic conductivityof the electrolyte depends on the cation-anion interactions. Ion pairs with a lower cation-anion interaction energies are expected to dissociate more, leading to an increased number of effective charge carriers. Calculated dissociation energies of the most stable ion pairs decreases as salts change from NaPF_6_ > NaPCPI > NaTIM > NaTCP, as shown in Supporting Figure 13 and Supporting Table 3. Ion pair dissociation energies for these Na-salts are 485, 443, 420 and 407 kJ mol^−1^, respectively. Therefore, improved ionic conductivity values for Na-salts with Hückel-type anions (TCP^−^ and TIM^−^) dissolved in PEO, compared to NaPF_6_, is expected and observed, due to a negative charge diffused over the entire anion via conjugated π bonds and electron-withdrawing cyano substituents.

Furthermore, attention should be directed at how the anion’s molecular structure affects the polymer matrix. A bulkier anion likely acts as a plasticizing agent, reducing the crystallinity of the polymer matrix. Inhibiting the crystallinity of the polymer grants more flexibility of the matrix and mobility of the charge carriers. The formation of tetrameric units of TCP^−^ anions via non-covalent π-π stacking is significant in that these units are bulkier than “free” anions; which can potentially plasticize the PEO matrix. Therefore, the “liquid-like” ionic conductivity of over 1 mS cm^−1^ for PEO_16_NaTCP above 70 °C is attributed to its low ion-pair interaction energies and anion-anion π-π stacking ability. Coupled with suitable electrochemical stability windows, these results demonstrate the potential applications of percyano Hückel-type anions in all-solid, sodium-ion battery electrolytes. Additionally, and according to Supporting Table 4, electrolytes based on PEG-500 present the same dependence.

### Thermal characterization of polymer electrolytes

Thermogravimetric (TG) experiments were performed to assess the thermal stabilities of pure salts. NaPCPI, NaTCP and NaTIM are found to be extremely stable, showing no significant weight losses up to 600, 540 and 570 °C, respectively (Supporting Figures 14, 15, 16). TG-IR characterization was performed to determine the decomposition products *in-situ*. All salts show significant evolution of CO_2_ above 550 °C and HCN above 600 °C (Supporting Figures 17–22). Additionally, CO is formed after heating NaTIM above 750 °C. NaPF_6_ is reported to undergo structural decomposition above 140 °C, accompanied by the release of PF_5_ gas^33^. Therefore, the fluorine-free salts are more thermally stable than NaPF_6_. While the salts tested show high thermal stabilities, the limit of the electrolyte operating temperature will be related to the solvent used; for example, PEO possesses a flash point of 229 °C^34^.

**Figure 6:**
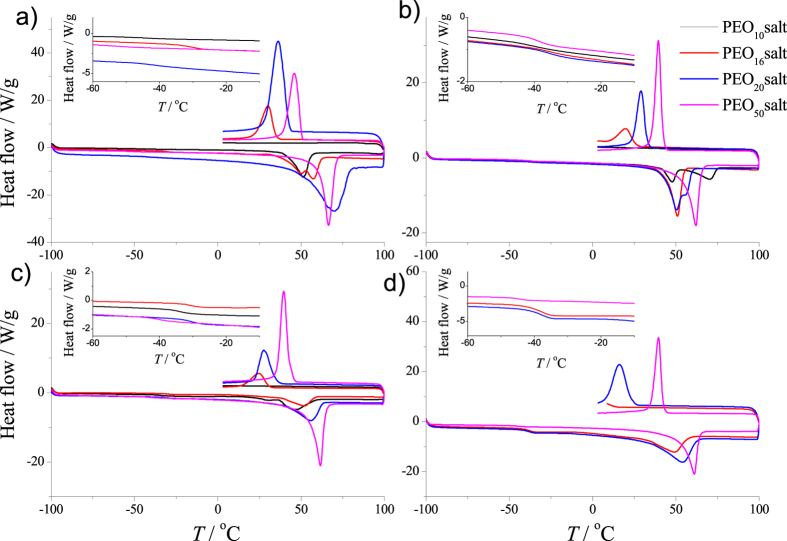
Differential scanning calorimetry curves of solid-polymer electrolytes. Experimental DSC curves of (**a**) NaPF_6_, (**b**) NaPCPI, (**c**) NaTCP and (**d**) NaTIM as a function of polymer concentration.

Differential scanning calorimetry (DSC) was completed on solid-polymer electrolytes with O:Na molar ratios of 10:1, 16:1, 20:1 and 50:1 (Supporting Table 5). DSC analysis provides insight into the polymer crystalline phase by assessing endothermic melting (*T*_m_) events. According to Fig. 6, all membranes with high polymer content (O:Na equal to 50:1) undergo a melting event above 61 °C; corresponding to the melting of crystalline PEO phase, as its accepted *T*_m_ is 65 °C^35^. As the concentration of salt increases to 20:1 and above, two phase melts are observed for membranes doped with NaPF_6_ and NaPCPI, suggesting the formation of apolymer-salt complex. Increasing the doping levels of Na-saltswill effectively decrease the amount the pure PEO. Crystalline complexes of PEO with sodium salts exhibit melting temperature above 70 °C^23^,^24^. Therefore, of the two melting events, the higher melting point is attributed to the polymer-salt complex and the lesser is due to the crystalline PEO phase.

To account for the rigidity of the polymer matrix, a glass transition temperature (*T*_g_) was also determined from DSC experiments. As salt content increases from PEO_50_NaPF_6_ to PEO_16_NaPF_6_, *T*_g_ increases from −53.9 to −29.4 °C (Supporting Table 5). In regards to membranes doped with NaPCPI, the glass transition temperature did not change significantly with respect to salt’s concentration, averaging at approximately *T*_g_ = −37.7 ± 2.2 °C over all membranes. A change in *T*_g_ with respect to salt concentration correlates to the anion’s ability to plasticize the polymer matrix. Since the incorporation of NaPCPI did not significantly affect *T*_g_, PCPI^−^ anion did not promote a loss of rigidity of PEO like PF_6_^−^.

PEO membranes doped with Hückel-type anions (TCP^−^, TIM^−^), according to Fig. 4, exhibit characteristic *T*_m_ and *T*_g_ during DSC analysis. PEO_10_NaTCP shows two*T*_m_ below 50 °C, corresponding to the melting of pure crystalline PEO and a polymer-salt complex phases. PEO_10_NaTIM was not characterized as the salt was not soluble at such a concentration. Membranes doped with NaTCP and NaTIM (greater than O:Na molar ratio of 10:1) undergo a single *T*_m_ which is associated with the melting of crystalline PEO. According to Supporting Table 5, PEO_50_NaTCP and PEO_50_NaTIM show *T*_g_ = −39.5 and −44.8 °C, respectively. At higher doping concentrations, *T*_g_ decreases to −30.7 and −37.8 °C for PEO_16_NaTCP and PEO_16_NaTIM.

Unlike membranes doped with PCPI^−^, TCP^−^ and TIM^−^ anions plasticize the polymer due to the fact that the glass transition temperature decreases with increasing salt concentration. Therefore, the aromatic nature of Hückel-type anions grants more flexibility of the PEO matrix allowing for better mobility of the charge carriers; which is confirmed in ionic conductivity measurements. Specifically, the formation of tetrameric units of TCP^−^ anions via non-covalent π-π stacking is significant in that these units are bulkier than “free” anions; which have greater potential to plasticize the PEO matrix.

## Discussion

In this work, novel fluorine-free Na-salts were solvated in PEG-500 and a matrix of PEO to obtain liquid and solid polymer electrolytes for sodium-ion battery applications. Structural analysis provided insight into the formation of tetrameric units of percyano Huckel-type TCP^−^ anions via π-π stacking in a polymer matrix. EIS experiments determined “liquid-like” ionic conductivities of over 1 mS cm^−1^ for PEO_16_NaTCP above 70 °C. The source of high performance is attributed to NaTCP’s low cation-anion interaction energy and its ability to plasticize the PEO matrix through the non-covalent stacking of its anions; reducing the rigidity of the polymer and allow for better mobility of charge carriers. Coupled with suitable electrochemical and thermal stability windows, these results demonstrate the potential applications of percyano Hückel-type anions in all-solid, sodium-ion battery electrolytes.

## Methods

### Structural characterization

Nuclear magnetic resonance (NMR) spectra were recorded on Varian Mercury 400 MHz and Bruker AVANCE 500 MHz.

Infrared (IR) absorption spectra were recorded on a Perkin-Elmer System 2000 FT-IR spectrometer with a wavenumber resolution of 1 cm^−1^. Fourier Transform Infrared Spectroscopy (FTir) studies were performed at room temperature.

Raman spectra were collected using a Nicolet Almega spectrometer. Two excitation lasers of 532 and 780 nm wavelengths were used. Only membranes with polymer to salt ratios of 16:1 and 10:1 were characterized in this manner.

Mass spectra were recorded with a Micro-mass ESI Q-TOF spectrometer.

### Synthesis of sodium 2,4,5-tricyanoimidazolate (NaTIM)

To a mixture of 2-amino-4,5-dicyanoimidazole (4.32 g, 32.45 mmol), distilled water (90 mL) and concentrated hydrochloric acid (36 mL), sodium nitrite (2.52 g, 36.53 mmol) was added portionwise. The precipitated diazonium salt was filtered off, washed with a small amount of distilled water and added to a previously prepared solution of sodium cyanide (3.33 g, 67.94 mmol) and copper cyanide (3.90 g, 43.55 mmol) in distilled water (300 mL). The content of flask was stirred for 0.5 h at room temperature and then tetraethylammonium bromide (25.23 g, 120.05 mmol) was added portionwise for 3 h. The resulting mixture was stirred overnight, after that time it was passed through the Celite^®^, concentrated under reduced pressure to approximately half its volume and extracted with methylene chloride (10 × 150 mL). After evaporation of the solvent a brown tetraethylammoniumtricyanoimidazolate was obtained. Crude product was purified by boiling with charcoal (2 g) in water (40 mL). The activated carbon was removed by hot gravity filtration. Subsequently, the filtrate was cooled: the precipitated solid was separated and dissolved in distilled water (235 mL). The resulting solution was acidified to pH = 1 with 12% HCl, extracted with diethyl ether (10 × 25 mL) and the combined ether fraction was dried over MgSO_4_. The filtration of the drying agent and evaporation of the solvent gave an almost white solid (1.21 g). In the next step acetonitrile (65 mL) was added to that product and the insoluble solid was separated out. The filtrate was treated with Na_2_CO_3_ (0.99 g) and stirred for 12 h. After that time activated carbon (0.5 g) was added and the mixture was stirred for 1 h at room temperature. Filtration off of the charcoal, evaporation of the solvent and washing the resulting solid with diethyl ether (3 × 10 mL) gave sodium 2,4,5-tricyano-1,3-imidazolate (1.35 g, 25%) as a white solid (Supporting Figure 23a).

^13^C NMR (100 MHz, CD_3_CN) *δ*/ppm 121.70, 116.19, 114.14, 103.94

HRMS (ESI^−^, *m*/*z*): [M]^−^_calcd_ = 166.0165, [M]^−^_found_ = 165.9827

### Synthesis of sodium 2,3,4,5-tetracyanopirolate (NaTCP)

In a flask equipped with a drying tube with CaCl_2_, tetracyano-1,4-dithiin (6.49 g, 30.00 mmol), sodium azide (1.96 g, 30.08 mmol) and ethanol (120 mL) were placed. The resulting mixture was stirred for 12 h, after this time the sulfur precipitate was filtered off and the filtrate was concentrated to dryness under reduced pressure. Afterwards the residue was treated with distilled water (120 mL) and stirred for 20 min at room temperature. The precipitated brown solid was filtered off and activated carbon (4 g) was added to the filtrate. The mixture was stirred for 5 h at room temperature. After filtering off the carbon, the aqueous solution was extracted successively with diethyl ether (5 × 40 mL) and then with ethyl acetate (10 × 40 mL). The collected acetate fractions were dried over MgSO_4_, afterwards the drying agent was filtered off and the filtrate was concentrated to dryness. The crude product was yield-purified by column chromatography (silica gel, gradient mixtures of acetonitrile-toluene − 1:3, 1:2, 1:1(v/v)). Subsequently, the crude product was dissolved in ethyl acetate, treated with activated carbon (2.5 g) and stirred overnight. After filtration of the charcoal and evaporation of the solvent, product was additionally purified by precipitation from the ethyl acetate solution by diethyl ether to give sodium 2,3,4,5-tetracyanopirolate (2.72 g, 48%)(Supporting Figure 23b).

^13^C NMR (100 MHz, CD_3_CN) *δ*/ppm: 133.83, 121,75, 117.13, 115.94;

HRMS (ESI^−^, *m*/*z*): [M]^−^_calcd_ = 142.0165, [M]^−^_found_ = 141.9628

### Synthesis of sodium pentacyanopropenide (NaPCPI)

Water (3.0 mL, 166.5 mmol) was added dropwise to a solution of tetracyanoethylene (20 g, 156.1 mmol) in pyridine (200 mL). The reaction mixture was heated at 100 °C for 15 min. Then the reaction mixture was poured into diethyl ether (1000 mL). Yellow precipitate was filtered off, washed with ether and dried in the air to give pyridiniumpentacyanopropenide (14.8 g, 77%) as a yellow solid (mp = 170–172 °C, lit. 167–168 °C^36^).

^1^H NMR (500 MHz; acetone-*d*_6_) *δ*/ppm: 14.11 (ws, 1 H, NH), 9.52 (m, 2 H, H-9,13), 8.82 (m, 1 H, H-11), 8.28 (m, 2 H, H-10,12)

^13^C NMR (125 MHz; acetone-*d*_6_) *δ*/ppm: 148.6 (1 C, C-11), 142.7 (2 C, C-9,13), 135.8 (1 C, C-2), 128.6 (2 C, C-10,12), 117.0 (2 C, C-6,7 or C-4,5), 114.7 (1 C, C-8), 114.1 (2 C, C-6,7 or C-4,5), 57.9 (2 C, C-1,3)

Sodium hydride (1.17 g, 48.7 mmol) was added slowly to a solution of pyridiniumpentacyanopropenide (8.0 g, 32.5 mmol) in dry tetrahydrofuran (100 mL) with cooling in an ice bath. The reaction mixture was stirred for 30 min and filtered. Filtrate was evaporated to dryness and then evaporated with toluene (3 × 50 mL) in order to remove pyridine. The yellow crude product was recrystallized from acetonitrile/toluene and chromatographed (100 g Al_2_O_3_ neutral, 500 mL acetonitrile as an eluent) to give sodium pentacyanopropenide (5.17 g, 92% yield) as a yellow solid (Supporting Figure 23c).

^13^C NMR (125 MHz; acetone-*d*_6_) *δ*/ppm: 136.7 (1 C, C-2), 117.6 (2 C, C-6,7 or C-4,5), 115.1 (1 C, C-8), 114.5 (2 C, C-6,7 or C-4,5), 58.3 (2 C, C-1,3)

15 N NMR (50 MHz; acetone-*d*_6_) *δ*/ppm: 279.5, 273.9, 261.3

### Single Crystals Preparation

Crystals of 12-crown-4 solvates with NaPCPI, NaTCP and NaTIM salts were obtained from solution containing ~20 mg of corresponding sodium salt and ~40 mg of 12C4 (molar ratio: 1:2.5). The mixtures prepared in hermetic glass vial were stirred and heated up to ~80 °C and then allowed to cool slowly to the room temperature, resulting in a single crystals.

Raman (Na(12C4)_2_^+^ PCPI^−^) 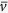/cm^−1^: 2246, 2215, 2191, 1484, 1443, 1393, 1304, 1294, 1112, 1095, 1047, 1032, 907, 855, 794;

Raman (Na(12C4)_2_^+^ TCP^−^) 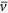/cm^−1^: 2229, 2225, 1474, 1414, 1308, 1114, 1095, 1076, 1047, 1027, 904, 855, 793.;

Raman (Na(12C4)_2_^+^ TIM^−^) 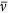/cm^−1^: 2235, 2227, 1459, 1374, 1339, 131, 1297, 1285, 1116, 1047, 1008, 901, 852, 793.

### X-ray crystallography

Selected single crystals of 12-crown-4 solvates were mounted in inert oil and transferred to the cold gas stream of the diffractometer. Diffraction data were measured at 120.0(1) K with mirror monochromatedCu*K*α or graphite monochromatedMo*K*α radiation on an Oxford Diffraction κ-CCD Gemini A Ultra diffractometer. Cell refinement and data collection as well as data reduction and analysis were performed with the CRYSALISPRO software. [CRYSALIS^PRO^ Software system, Agilent Technologies, Oxford, UK, 2014]. Structures were solved by direct methods using the SHELXT^37^ structure solution program and refined by full-matrix least-squares against *F*^2^ with SHELXL-2014^38^ and OLEX2^39^ programs. The crystal data and experimental parameters are summarized in Supporting Table 1. CCDC1425674–1425676 entries contain the supplementary crystallographic data for this paper. Single crystal of Na(12C4)_2_^+^ PCPI^−^ exhibit structural disorder. PCPI^−^ anions are disordered over two positions with occupancy ratios of 0.896(4):0.104(4). The non-merohedric crystal domains of Na(12C4)_2_^+^ TCP^−^ were found to be twinned in the triclinic space group *P*1, with a domain ratio of 0.8498(4):0.1502(4) in the final refinement. These data can be obtained free of charge from The Cambridge Crystallographic Data Centre via www.ccdc.cam.ac.uk/data_request/cif.

### Crystal structure determination

Crystal Data for Na(12C4)_2_^+^ PCPI^−^: C_24_H_32_N_5_NaO_8_ (*M* = 541.53 g/mol), triclinic, *P*1;^−^, *a* = 7.8139(2) Å, *b* = 13.9616(6) Å, *c* = 14.0604(6) Å, *α* = 119.144(5)°, *β* = 94.331(3)°, *γ* = 94.794(3)°, *V* = 1323.08(10) Å^3^, *Z* = 2, μ(CuKα) = 1.000 mm^−1^, 19779 reflections measured (7.264° ≤ 2Θ ≤ 133.798°), 4681 unique (*R*_int_ = 0.0334, R_sigma_ = 0.0252) which were used in all calculations. The final *R*_1_ was 0.0299 (*I* > 2*σ(I*)) and *wR*_2_ was 0.0770 (all data).

Crystal Data for Na(12C4)_2_^+^ TCP^−^: C_24_H_32_N_5_O_8_Na (*M* = 541.53 g/mol), triclinic, *P*1;^−^, *a* = 14.8918(3) Å, *b* = 16.1844(3) Å, *c* = 23.6019(5) Å, *α* = 88.8849(17)°, *β* = 75.7581(19)°, *γ* = 78.8007(18)°, *V* = 5406.2(2) Å^3^, *Z* = 8, μ(CuKα) = 0.979 mm^−1^, 35861 reflections measured (6.244° ≤ 2Θ ≤ 134.948°), 35861 unique (*R*_int_ = 0.0390, R_sigma_ = 0.0500) which were used in all calculations. The final *R*_1_ was 0.0492 (*I* > 2*σ(I*)) and *wR*_2_ was 0.1394 (all data).

Crystal Data for Na(12C4)_2_^+^ TIM^−^: C_22_H_32_N_5_NaO_8_ (*M* = 517.51 g/mol), monoclinic, *P*2_1_/*n, a* = 8.4422(2) Å, *b* = 13.7081(3) Å, *c* = 22.2632(5) Å, *β* = 94.506(2)°, *V* = 2568.47(10) Å^3^, *Z* = 4, μ(MoKα) = 0.116 mm^−1^, 60234 reflections measured (6.554° ≤ 2Θ ≤ 53.546°), 5487 unique (*R*_int_ = 0.0577, R_sigma_ = 0.0264) which were used in all calculations. The final *R*_1_ was 0.0340 (*I* > *2σ(I*)) and *wR*_2_ was 0.0815 (all data).

### Solid-polymer electrolyte preparation

High molecular weight poly(ethylene oxide) (PEO, Sigma Aldrich *M*_n_≈ 4,000,000) was complexed with synthesized salts to produce polymer electrolytes (also referred to as “membranes”). NaPF_6_ was obtained from Aldrich (98%) and was dried before use. The abbreviation O:Na is used throughout the paper to indicate the ratio of [CH_2_CH_2_O] repeating units to moles of salt. Membranes were prepared using O:Na molar ratios of 10:1, 16:1, 20:1 and 50:1. All precursor materials were dried for one week to remove the influence of moisture contamination. Samples were prepared in a moisture-free glove box, by mixing components using a mortar and pestle. Then, they were put into argon-filled coffee bags and hot-pressed. Hot-pressing consisted of applying a pressure of 17.7 kg cm^−2^ for 15 minutes, then 141.5 kg cm^−2^ for 45 min; both at 90 °C.

### Thermal characterization

Differential Scanning Calorimetry (DSC) testing was performed using a TA Instruments Q200 calorimeter under flowing nitrogen (25 mL min^−1^) at a heating rate of 10 °C min^−1^from −100 to 100 °C. Samples were contained in covered pans made of aluminum and made by the same company. Thermogravimetric (TG) IR experiments were completed using a NICOLET 6700 TG-FTIR at a heating rate of 10 °C min^−1^ under inert atmospheric conditions.

### Electrochemical stability

The electrochemical stability windows of the electrolytes were determined by cyclic voltammetry (CV). In order to test PEO-membranes, a two-electrode cell was used. This setup consisted of glassy carbon electrode, membrane and sodium metal as reference. Membranes were tested at 50 °C. A three-electrode cell was used to investigate liquid electrolytes (PEG-based). Three-electrode cell assembly consisted of tantalum wire (counter electrode), electrolyte soakedglassy fiber membrane and glassy carbon (working electrode); a sodium metal was used as the reference electrode. Liquid electrolytes were tested at 25 °C. Scans were performed at 0.1 mV s^−1^ rates using a VMP3 potentiostat from Bio-logic Science Instruments.

### Ionic conductivity measurements

The ionic conductivity was determined by AC impedance spectroscopy using either custom-made stainless steel cells for liquid electrolytes or aSwagelok-type cells for membranes. The cells were assembled in an argon-atmosphere drybox (<1 ppm H_2_O). Conductivity tests were performed by heating the samples in a Lauda E 300 thermostat. In regards to liquid electrolytes, samples were tested from −10 to 60 °C. For membranes, prior to conductivity measurements, cells were kept at 80 °C for 1 h in order to thermally condition the membrane, and then tested down to 20 °C. Samples containing the same salt and solvent, but at different concentrations, were tested at the same time. Impedance tests were performed by applying 5 mV amplitude signal from 500 kHz to 100 Hz using a VMP3 potentiostat from Bio-logic Science Instruments. Impedance data were evaluated by EC-lab software.

### Calculations

The presence of π-stacking interactions was examined by relaxed PES scan of distance between the centers of two TCP anion rings. Gas phase potential energy curves were evaluated at HF/jun-cc-pVDZ and MP2/jun-cc-pVDZ levels of theory. The dissociation energies (∆*E*_d_) were calculated as difference between energies of ion-pairs and separate ions, with BSSE correction. The B3LYP density functional and the 6–311 + G(d) basis set were used to optimize all geometries. The oxidation potentials (Δ*E*_v_) were calculated by adjustment vertical ionization potentials toward Na^+^/Na (−1.12 V), calculated at M06-2X/6-311 + G(d) level of theory. All computations were made using the Gaussian09 program^40^.

## Additional Information

**How to cite this article**: Bitner-Michalska, A. *et al*. Fluorine-free electrolytes for all-solid sodium-ion batteries based on percyano-substituted organic salts. *Sci. Rep.* **7**, 40036; doi: 10.1038/srep40036 (2017).

**Publisher's note:** Springer Nature remains neutral with regard to jurisdictional claims in published maps and institutional affiliations.

## Supplementary Material

Supplementary Information

## Figures and Tables

**Figure 1:**
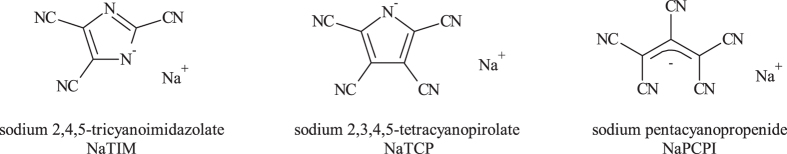
Structures of synthesized salts.

**Table 1:** Ionic conductivity values of PEO_16_NaPF_6_, PEO_20_NaPCPI, PEO_16_NaTCP and PEO_16_NaTIM membranes at various temperatures.

Temperature, °C	σ (NaPF_6_), mS cm^−1^	σ (NaPCPI), mS cm^−1^	σ (NaTCP), mS cm^−1^	σ (NaTIM), mS cm^−1^
20	0.0065	0.0014	0.019	0.0084
30	0.043	0.011	0.069	0.057
40	0.050	0.074	0.47	0.13
50	0.086	0.17	0.70	0.22
60	0.15	0.27	0.93	0.33
70	0.21	0.37	1.24	0.55
80	0.31	0.53	1.57	0.77
