# Supplementary material for: Fluorine-free electrolytes for all-solid sodium-ion batteries based on percyano-substituted organic salts
Source: Sci Rep. 2017 Jan 9;7:40036. doi: 10.1038/srep40036 (PMC5220368; doi:10.1038/srep40036)
Supplement: Supplementary Information [file srep40036-s1.pdf]

Supplementary Material

Fluorine-free electrolytes for all-solid sodium-ion batteries based on percyano-substituted organic salts

Anna Bitner-Michalska, Gene M. Nolis, Grażyna Żukowska, Aldona Zalewska, Marcin Poterała, Tomasz Trzeciak, Maciej Dranka, Michał Kalita, Piotr Jankowski, Leszek Niedzicki, Janusz Zachara, Marek Marcinek, Władysław Wieczorek *

Polymer Ionics Research Group

Faculty of Chemistry

Warsaw University of Technology

Noakowskiego 3, 00-664 Warsaw, Poland

X-ray crystallography

A suitable single crystals of $Na(12C4)_2^+PCPI^-$, $Na(12C4)_2^+TCP^-$ and $Na(12C4)_2^+TIM^-$ solvates were selected under the polarizing microscope and mounted in inert oil on an Oxford Diffraction κ-CCD Gemini A Ultra diffractometer. Crystals were kept at 120.0(1) K during data collection. Cell refinement and data collection as well as data reduction and analysis were performed with the CRYSALIS^{PRO} software. [1] Using Olex2 [2], the structure was solved with the ShelXT [3] structure solution program using Direct Methods and refined with the ShelXL [4] refinement package using Least Squares minimization. The crystal data and experimental parameters are summarized in Table 3. Hydrogen atoms were added to the structure model at a geometrically idealized coordinates and refined as riding atoms. Solvate $Na(12C4)_2^+$ $PCPI^-$ exhibit structural disorder. PCPI anions are disordered over two positions with occupancy ratios of 0.896(4):0.104(4). The non-merohedric crystal domains of $Na(12C4)_2^+$ TCP^- were found to be twinned in the triclinic space group $P\bar{1}$ with two domains of ratio 0.8498(4):0.1502(4) in the final refinement and the hklf 5 procedure was used for the refinement. Both orientation matrices identified using the program CRYSALIS^{PRO} are related by a twofold rotation around the [-0.2 0.08 0.02] direction in reciprocal space (0 1 0 in direct space).

Supporting table 1. Crystal Data for the Single Crystal X-ray Structures of 12C4 solvates of NaPCPI, NaTCP and NaTIM.

**Compound reference**	**$Na(12C4)_2^+$ $PCPI^-$**	**$Na(12C4)_2^+$ TCP^-**	**$Na(12C4)_2^+$ TIM^-**
Chemical formula	$C_{16}H_{32}NaO_8·C_8N_5$	$C_{16}H_{32}NaO_8·C_8N_5$	$C_{16}H_{32}NaO_8·C_6N_5$
Formula Mass	541.53	541.53	517.51
Crystal system	Triclinic	Triclinic	Monoclinic
a/ Å	7.8139(2)	14.8918(3)	8.4422(2)
b/ Å	13.9616(6)	16.1844(3)	13.7081(3)
c/ Å	14.0604(6)	23.6019(5)	22.2632(5)
α/°	119.144(5)	88.8849(17)	90
β/°	94.331(3)	75.7581(19)	94.506(2)
γ/°	94.794(3)	78.8007(18)	90
Unit cell volume/ Å^3	1323.08(10)	5406.2(2)	2568.47(10)
Temperature/K	120.0(1)	120.0(1)	120.0(1)
Space group	$P\bar{1}$	$P\bar{1}$	$P2_1/n$
No. of formula units per unit cell, *Z*	2	8	4
No. of reflections measured	19779	35861	60234
No. of independent reflections	4681	35861	5487
R_{int}	0.0334	0.0390	0.0577
Final R_1 values ($I > 2\sigma(I)$)	0.0299	0.0492	0.0340
Final $wR(F^2)$ values ($I > 2\sigma(I)$)	0.0725	0.1265	0.0747
Final R_1 values (all data)	0.0381	0.0746	0.0471
Final $wR(F^2)$ values (all data)	0.0770	0.1394	0.0815
Goodness of fit on F^2	1.026	0.957	1.035
CCDC number	1425674	1425675	1425676

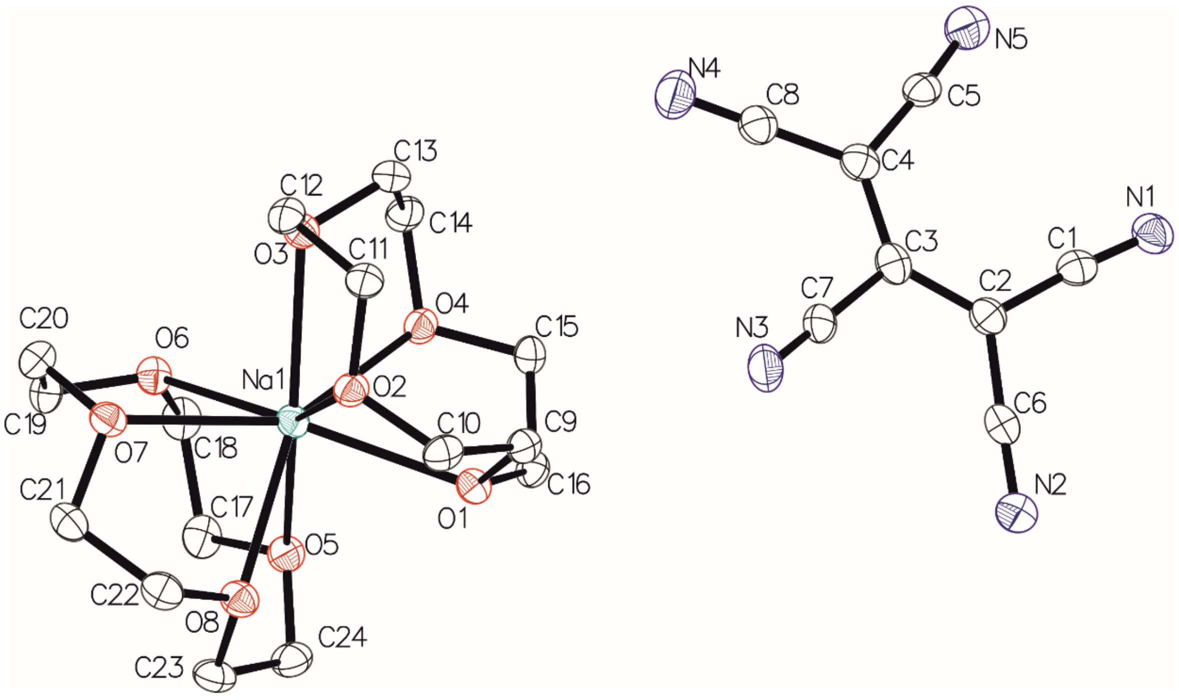

Supporting figure 1. Molecular structure of crystalline $Na(12C4)_2^+$ $PCPI^-$ solvate with atom numbering scheme. Hydrogen atoms and partial disorder of PCPI anion were omitted for clarity.

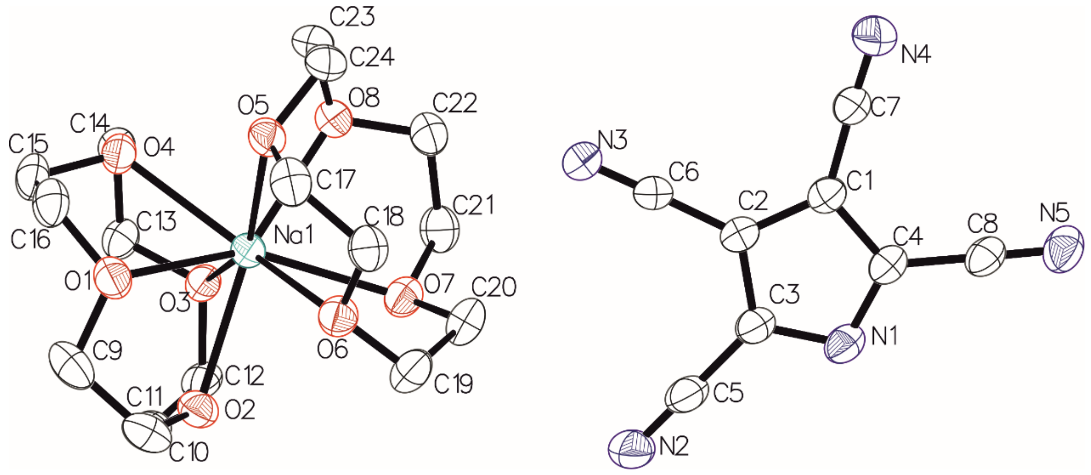

Supporting figure 2. Ortep plot of molecular assembly observed in $Na(12C4)_2^+$ TCP^- solvate. Hydrogen atoms and disorder of 12C4 rings were omitted for clarity.

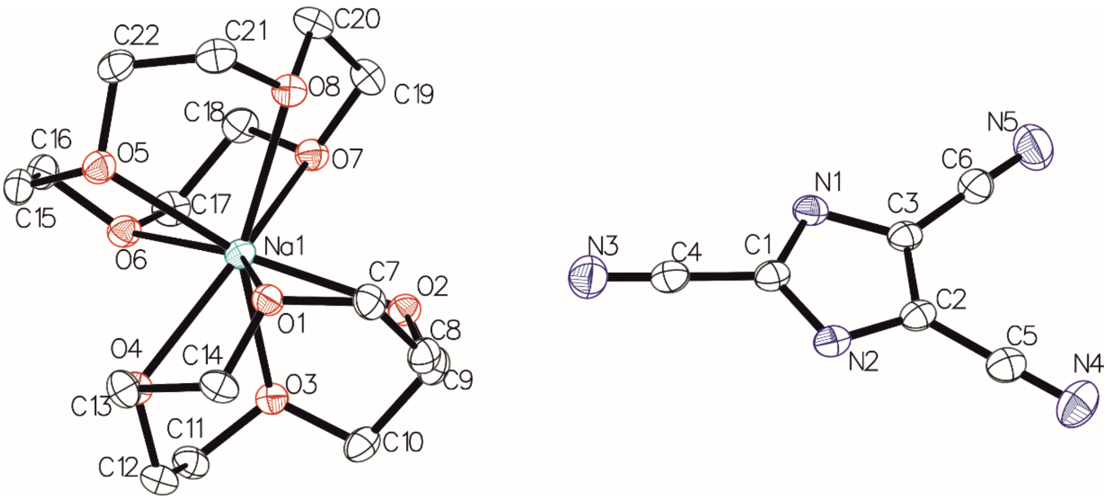

Supporting figure 3. Molecular structure of crystalline $Na(12C4)_2^+$ TIM^- solvate. Hydrogen atoms were omitted for clarity.

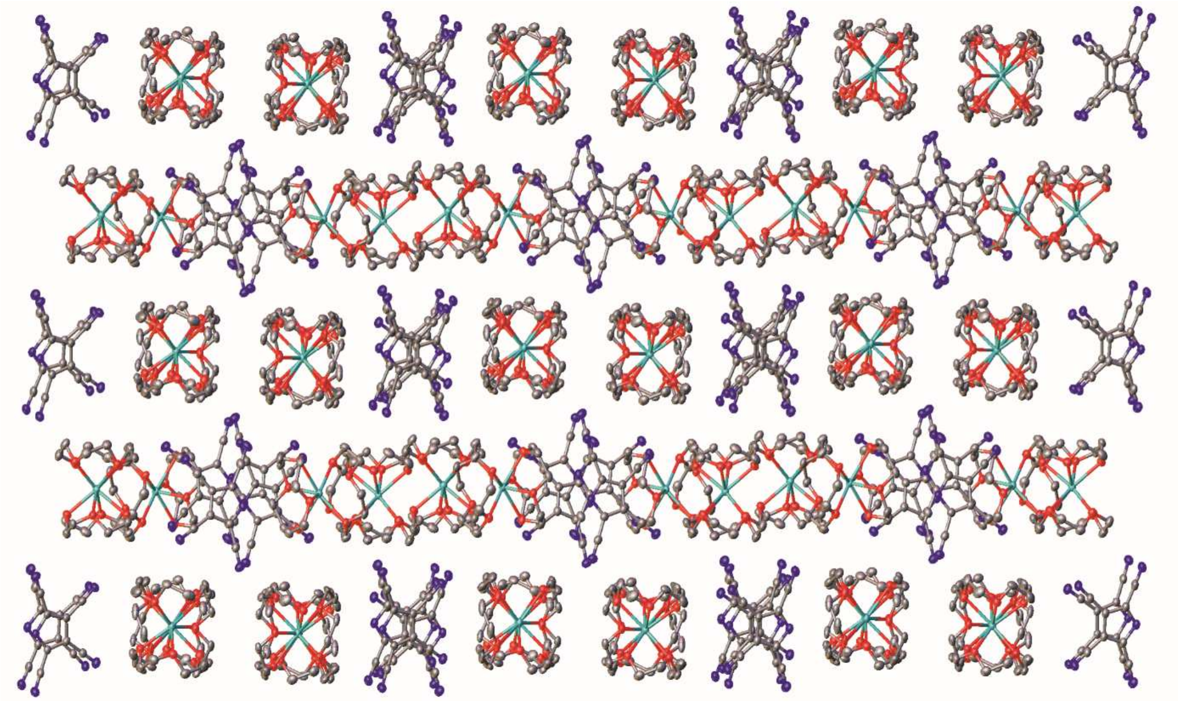

Supporting figure 4. Projection of the crystal packing in $Na(12C4)_2^+TCP^-$ along *a* axis showing columnar arrangement of anions and cations. Hydrogen atoms have been omitted for clarity. Color code: gray C; red O; blue: N, cyan: Na.

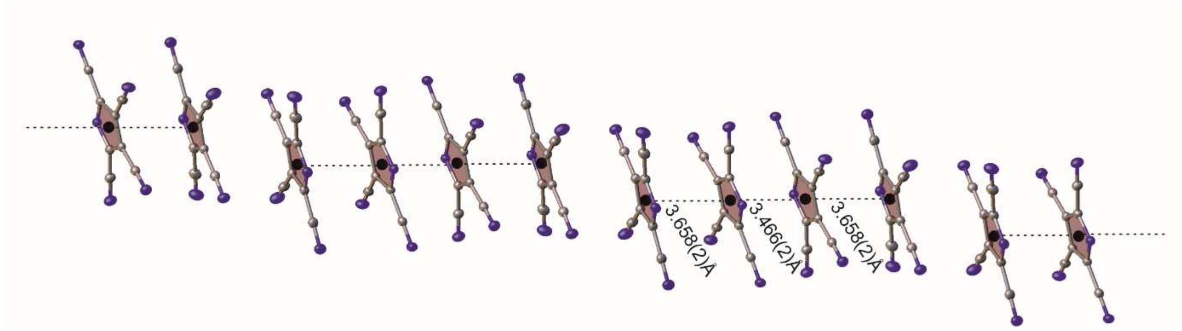

Supporting figure 5. Detailed view of columnar arrangement of anions in $Na(12C4)_2^{+}TCP^{-}$. Tetrameric units are formed via π–π stacking interaction between TCP rings with short centroid-centroid distance.

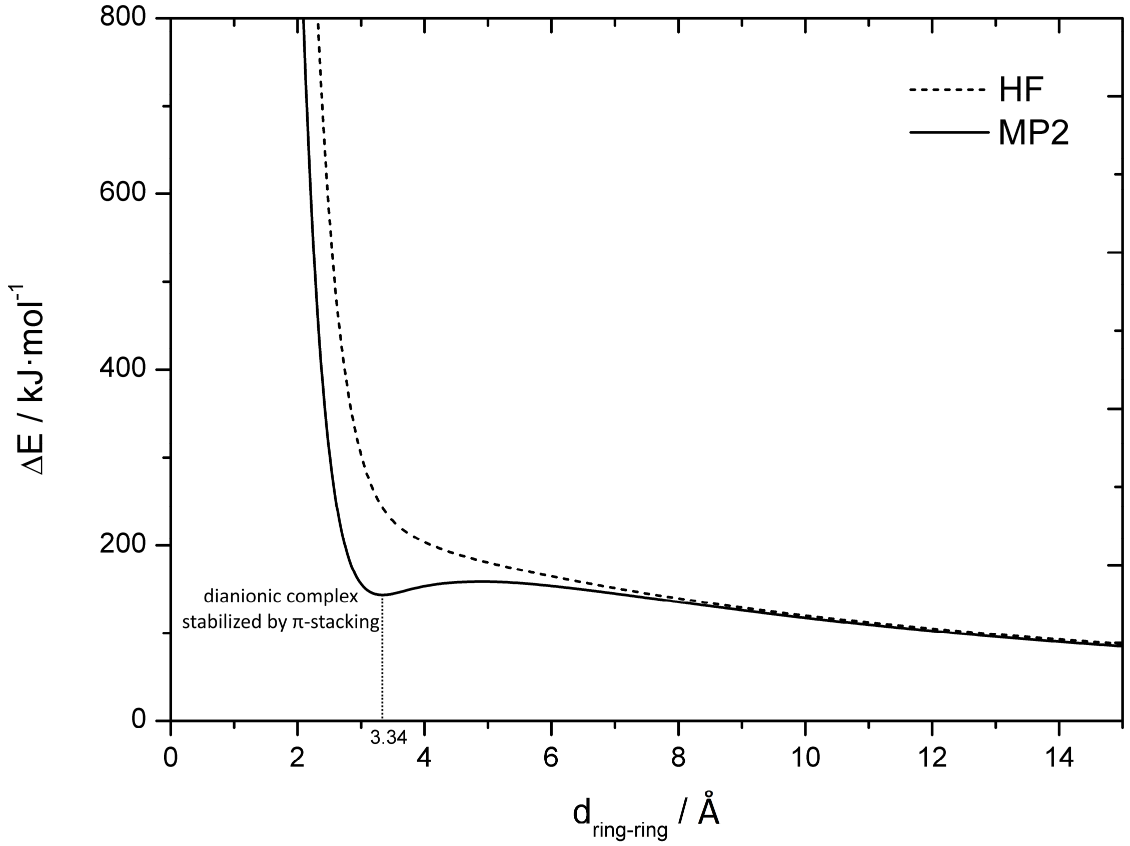

Supporting figure 6. BSSE-corrected interaction energies between two TCP anions, as a function of distance between the centers of rings; comparison of uncorrelated (HF/jun-cc-pVDZ) and correlated (MP2/jun-cc-pVDZ) methods in prediction of π-stacking interactions.

Supporting table 2. Detailed geometry of π-π contacts in $Na(12C4)_2^+TCP^-$.

Plane	a-b	b-c	c-d
**Centroid-to-plane distance/Å**	3.526(2)	3.347(2)	3.460(2)
**Centroid-to-centroid distance/Å**	3.658(2)	3.466(2)	3.658(2)
**Plane-to-plane angle /°**	5.06(9)	0.0(2)	5.06(9)
**Shift displacement distance/Å**	0.972(5)	0.901(5)	1.186(4)

plane **a**: [C2 C1 C4 N1 C3]; plane **b**: [C26 C27 C28 N6 C25]; plane **c**: [C26 C27 C28 N6 C25]^i, plane **d**: [C2 C1 C4 N1 C3]^i; symmetry code *i*=1-*x*, 1-*y*, 2-*z*;

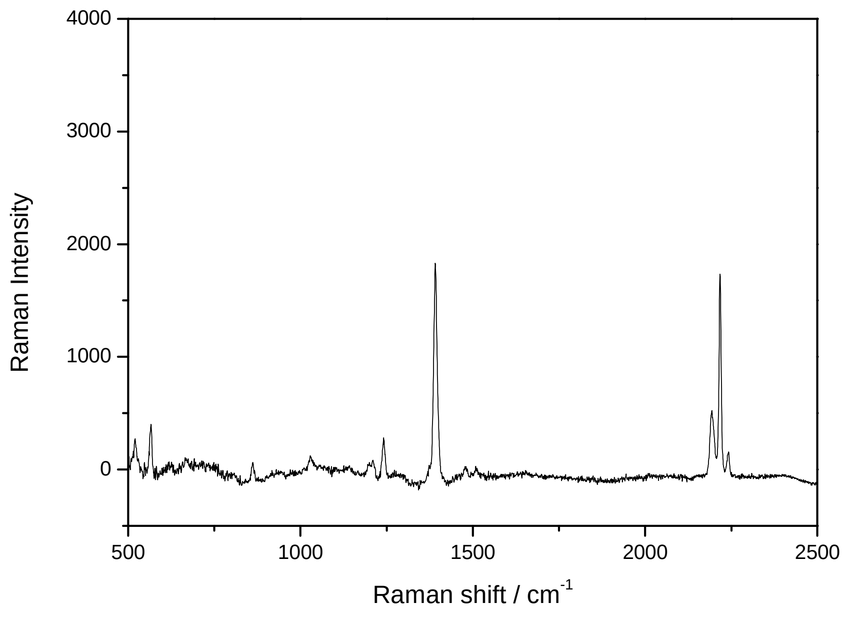

Supporting figure 7. $PEO_{10}PCPI$ Raman spectra.

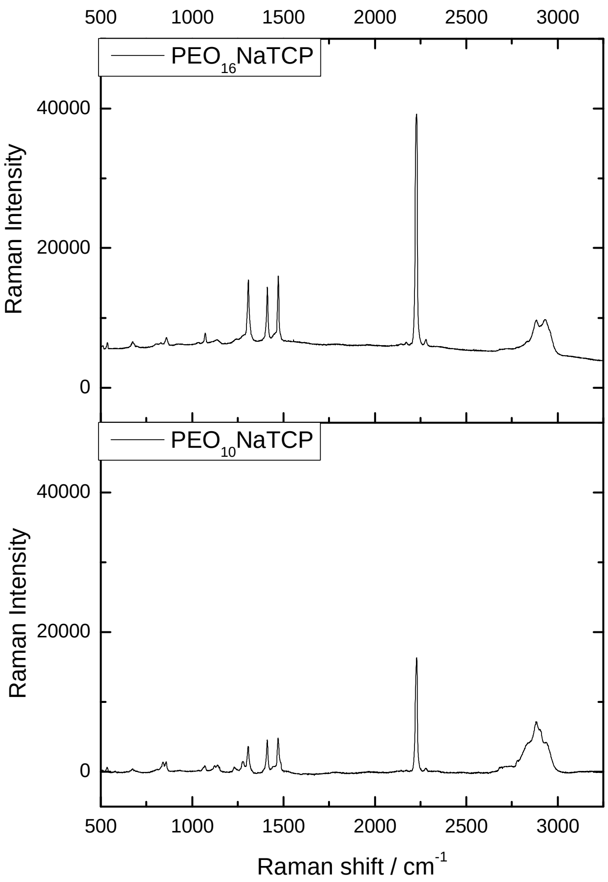

Supporting figure 8. $PEO_{10}NaTCP$ and $PEO_{16}NaTCP$ Raman spectra.

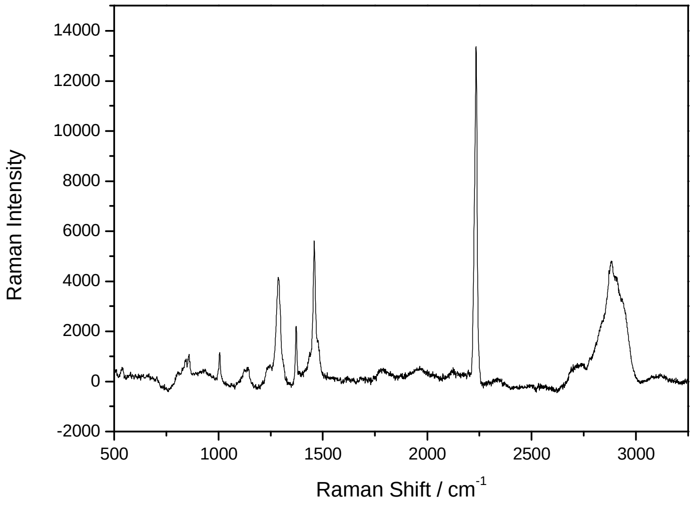

Supporting figure 9. $PEO_{16}NaTIM$ Raman spectra.

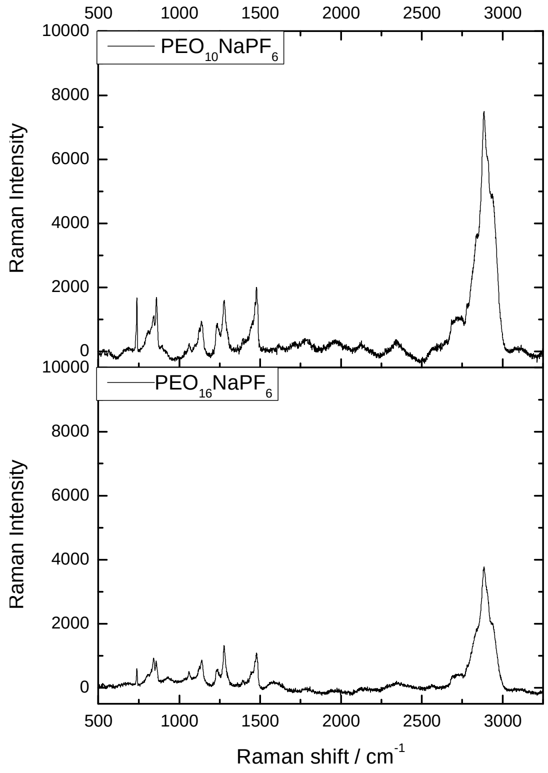

Supporting figure 10. $PEO_{10}NaPF_6$ and $PEO_{16}NaPF_6$ Raman spectra.

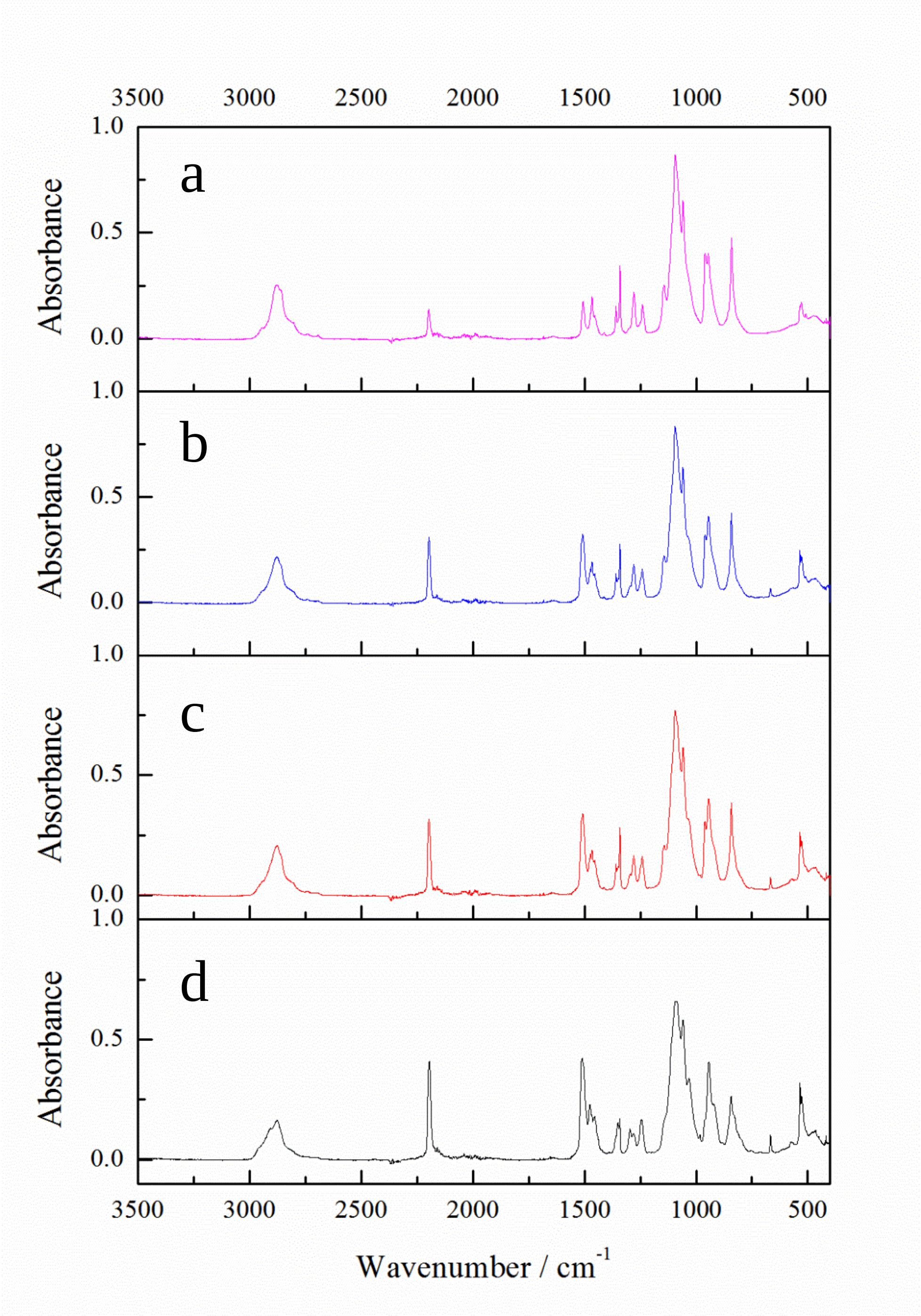

Supporting figure 11. FTIR spectra for PEO_{50}NaPCPI (a), PEO_{20}NaPCPI (b), PEO_{16}NaPCPI (c) and PEO_{10}NaPCPI (d).

Supporting table 3. Calculated dissociation energies of the most stable ion-pairs (ΔE_d) and anion oxidation potentials (ΔE_v).

anion	ΔE_d [kJ·mol^{-1}] B3LYP/6-311+G(d)	ΔE_v [V vs. Na^+/Na^0] M06-2X/6-311+G(d)
PCPI	443	3.25
TIM	420	3.68
TCP	407	3.89
PF_6^-	485	7.02
TFSI	490	5.45

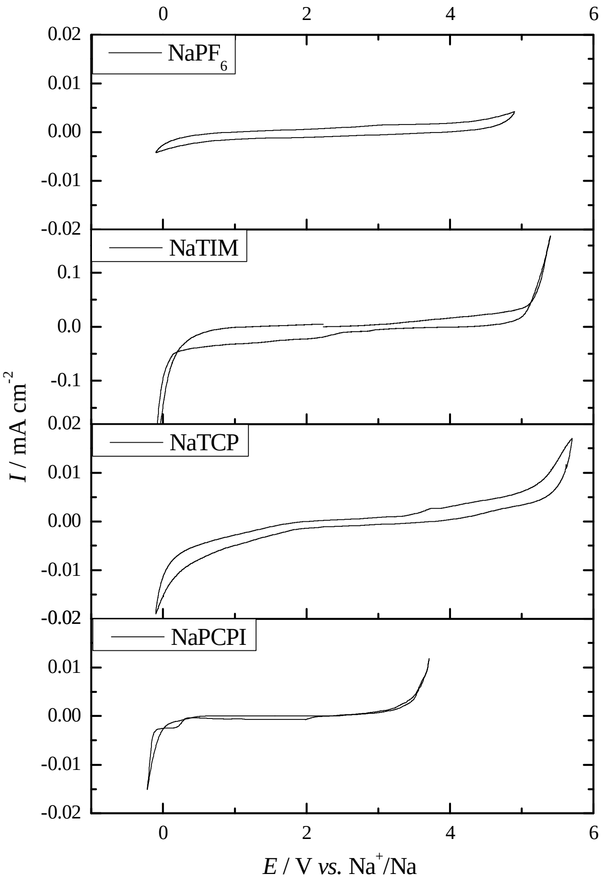

Supporting figure 12. Cyclic voltammetry of electrolytes (0.75 mol/kg of salts in PEG-500)

Supporting table 4. Ionic conductivity values of 0.75 mol kg^{-1} of NaPF6, NaPCPI, NaTCP and NaTIM in PEG at various temperatures.

Temperature, ˚C	σ ($NaPF_6$), mS cm^{-1}	σ (NaPCPI), mS cm^{-1}	σ (NaTCP), mS cm^{-1}	σ (NaTIM), mS cm^{-1}
**10**	0.35	0.48	0.66	0.77
**20**	0.57	0.75	1.05	1.16
**25**	0.71	0.94	1.24	1.36
**30**	0.84	1.14	1.52	1.57
**40**	1.17	1.56	2.16	2.18
**50**	1.56	2.09	2.90	2.83
**60**	1.98	2.67	3.77	3.53

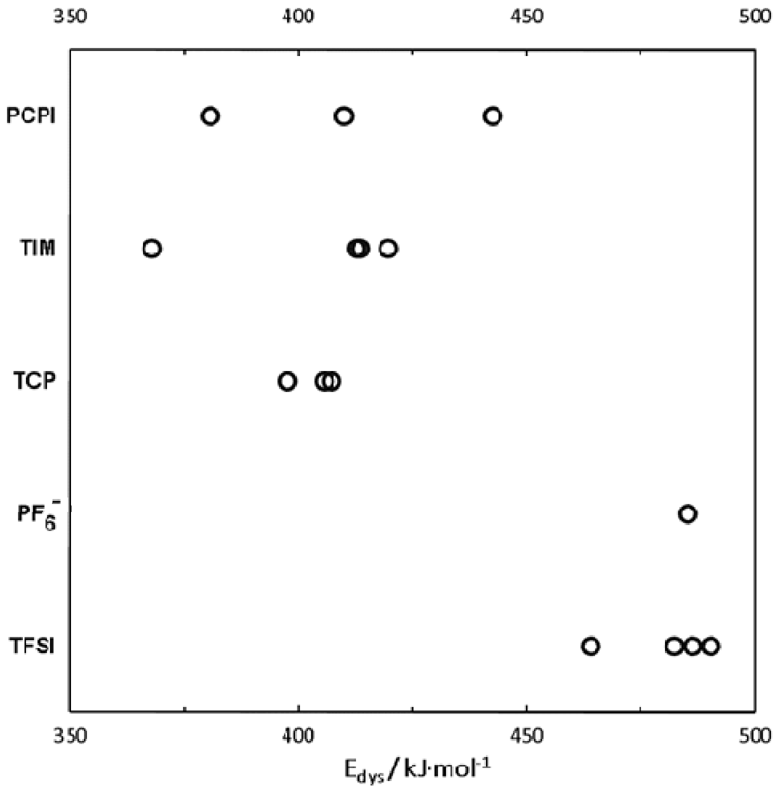

Supporting figure 13. Calculated dissociation energies for all found stable ion-pairs; B3LYP/6-311+G(d)

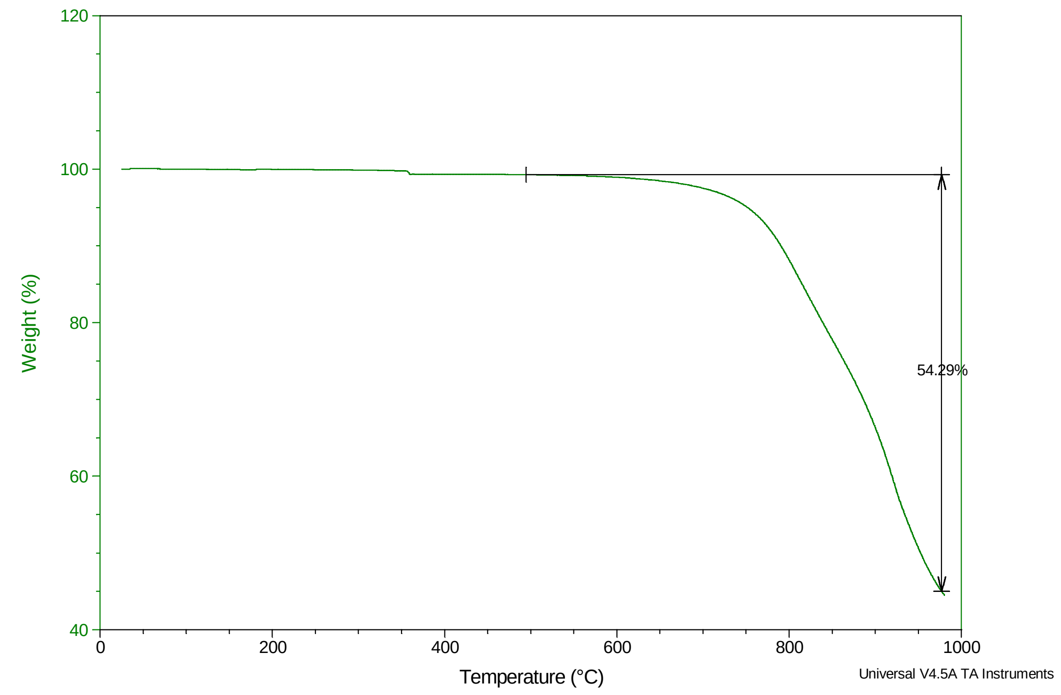

Supporting figure 14. TG of pure NaPCPI salt in inert atmosphere.

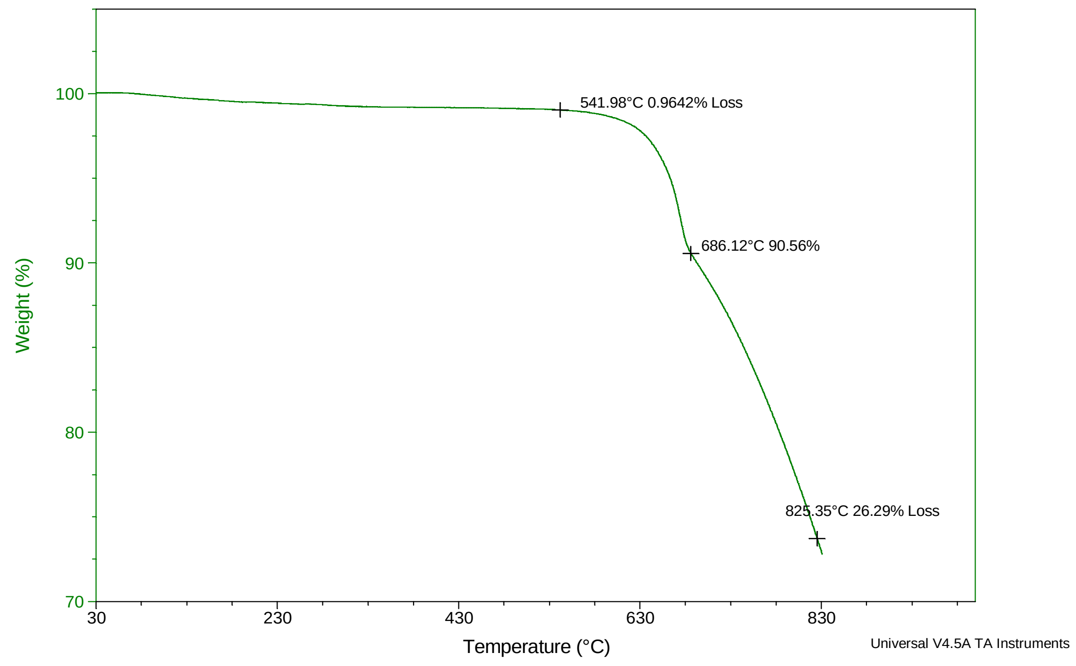

Supporting figure 15. TG of NaTCP heated at 10 ˚C/min in inert atmosphere.

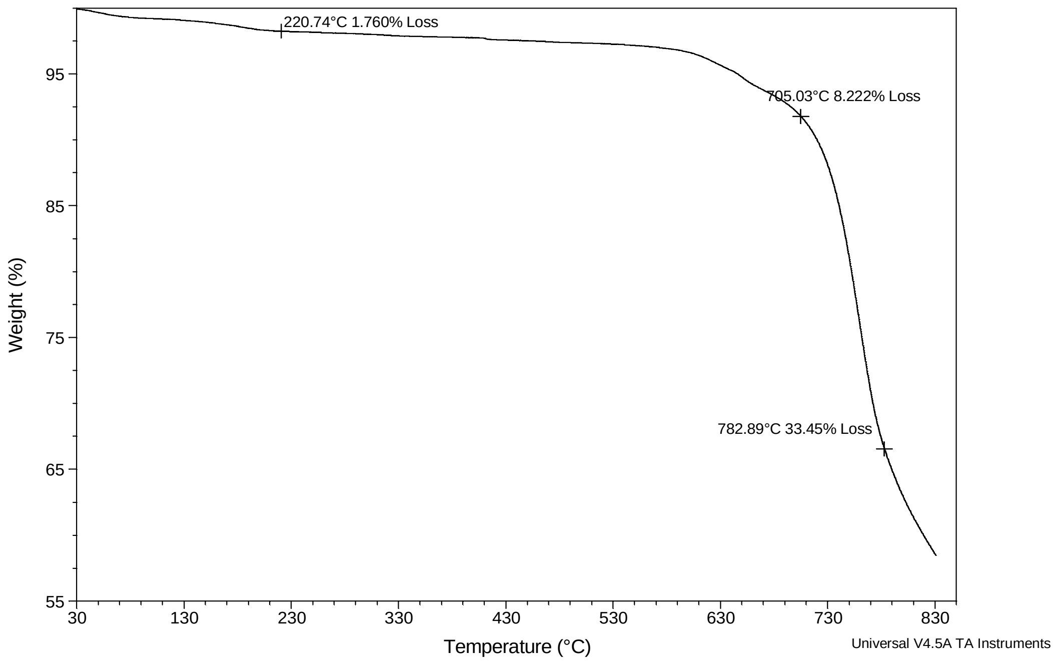

Supporting figure 16. TG of NaTIM heated at 10 ˚C/min in inert atmosphere.

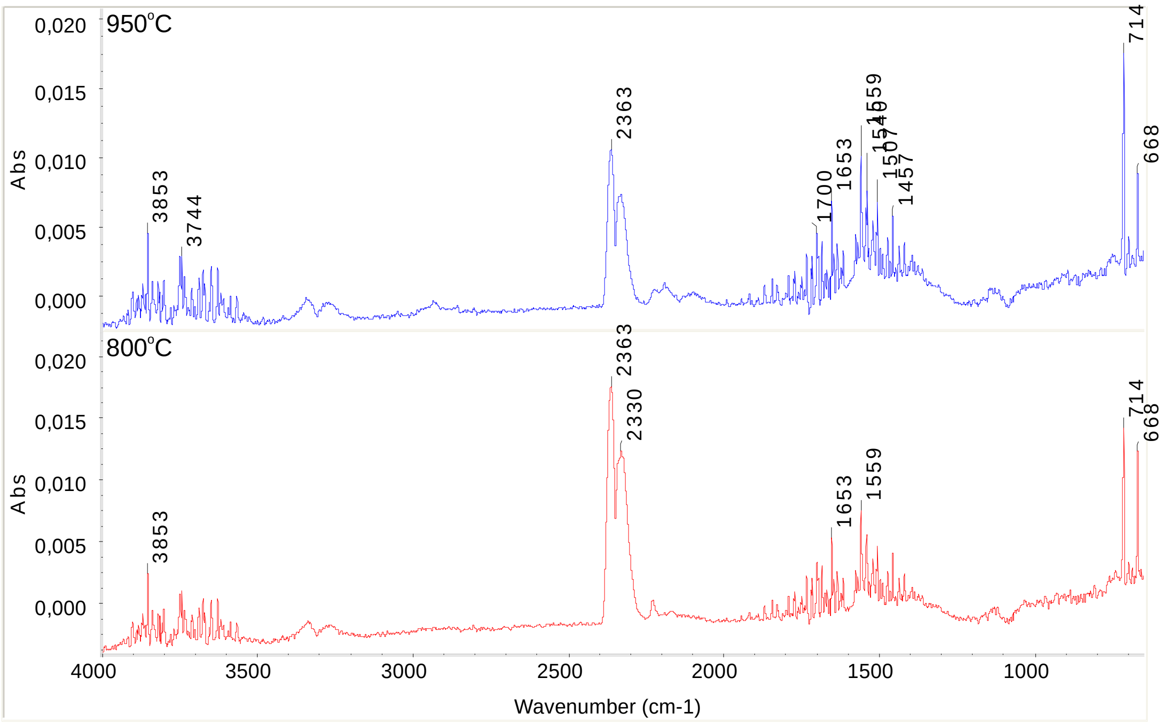

Supporting figure 17. FTIR spectra of NaPCPI at 800 ˚C (blue) and 950 ˚C (red).

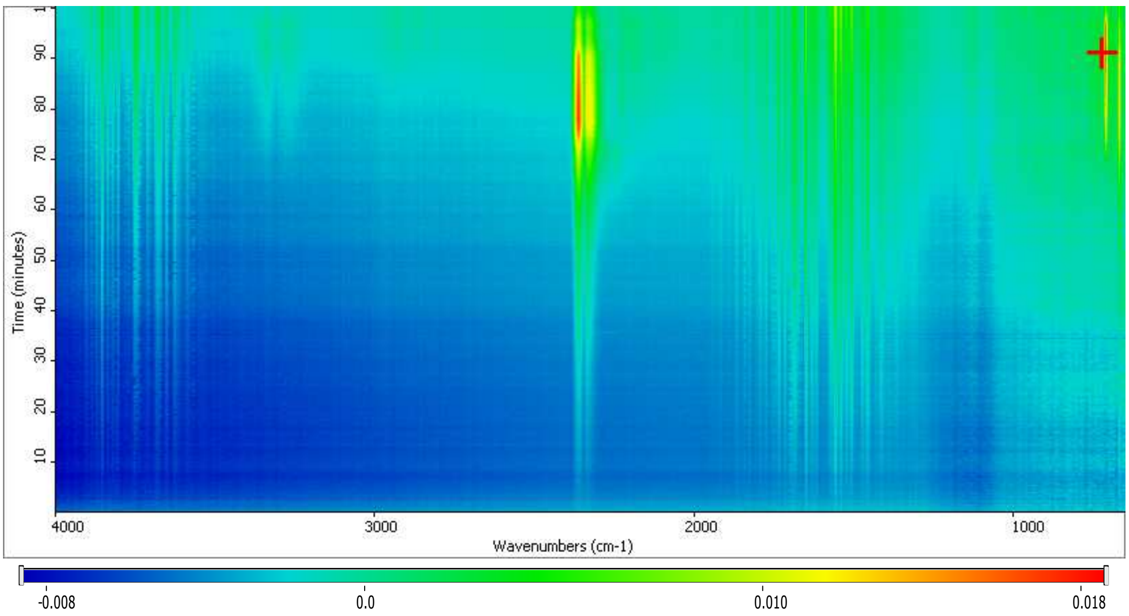

Supporting figure 18. FTIR spectra of evolved gases during TG of NaPCPI. The legend below the figure corresponds to peak intensity; where blue to red shift corresponds from negligible to intense peaks.

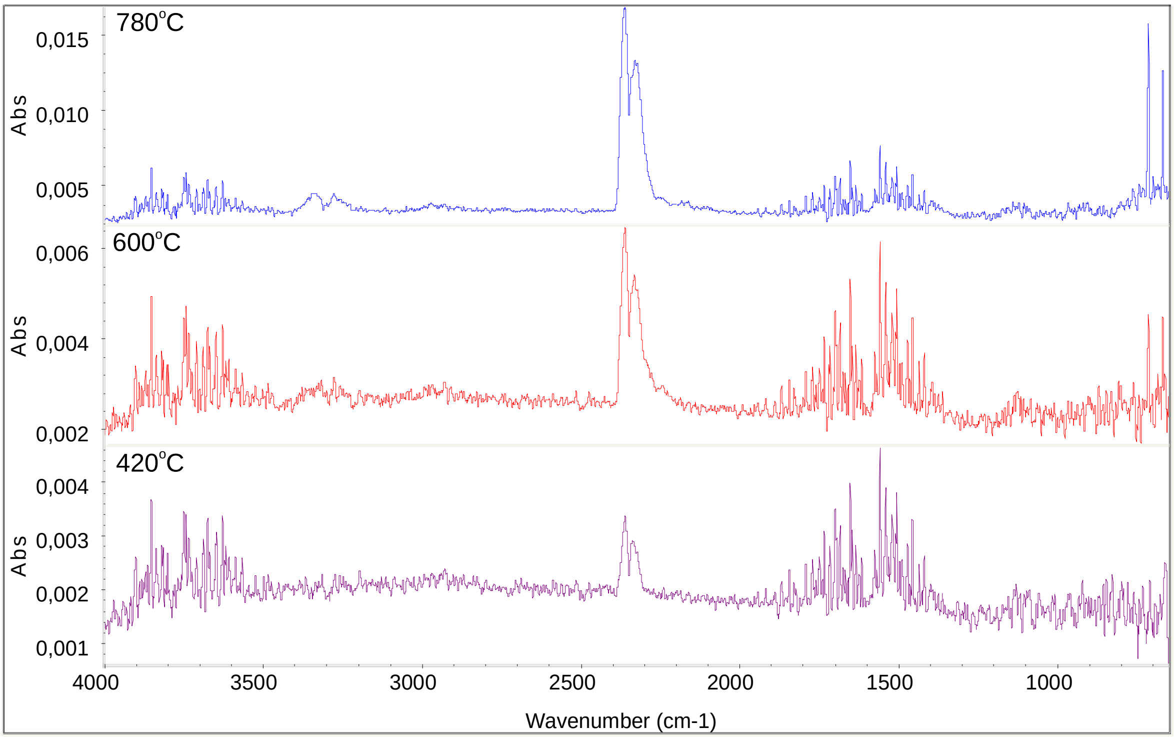

Supporting figure 19. FTIR spectra of evolved products of NaTCP at 420 (blue), 600 (red) and 780˚C (purple).

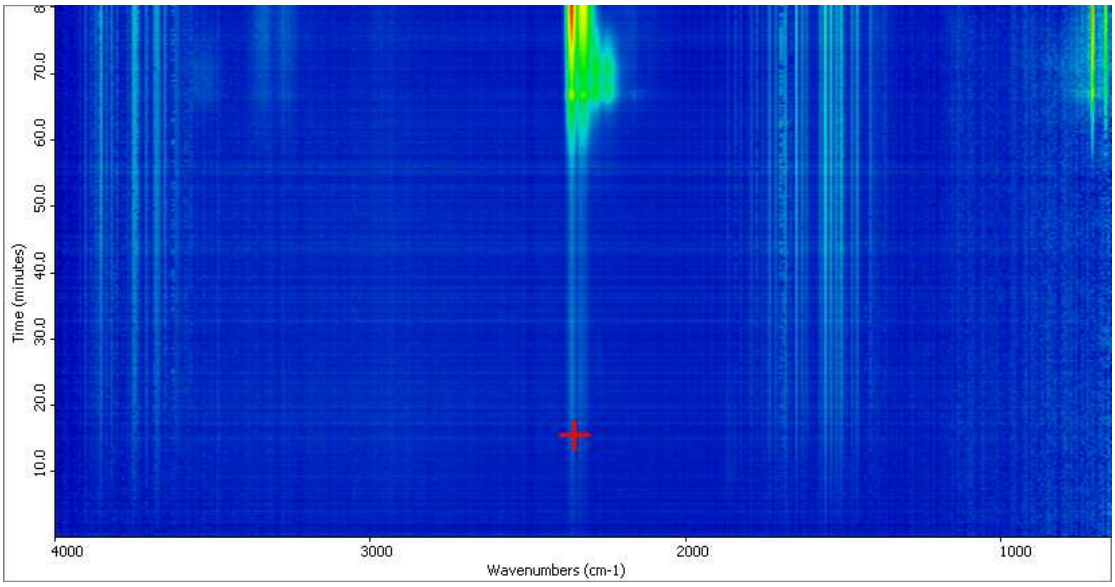

Supporting figure 20. FTIR spectra of evolved gases during TG of NaTCP. The legend below the figure corresponds to peak intensity; where blue to red shift corresponds from negligible to intense peaks.

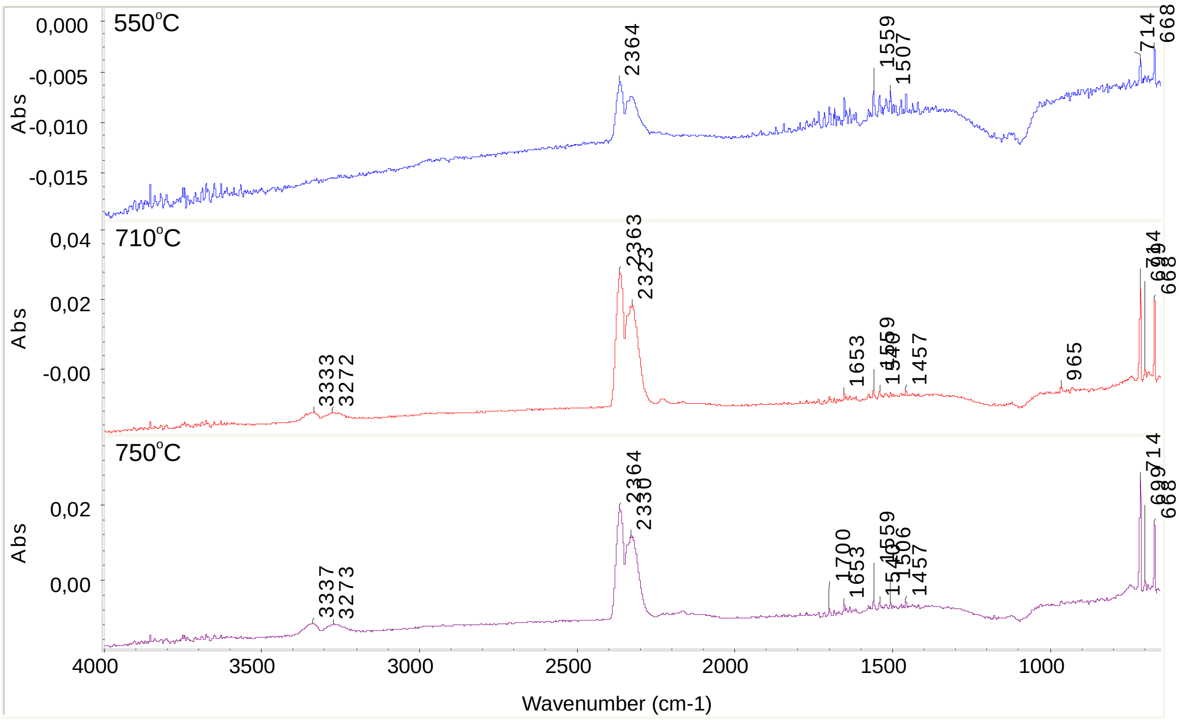

Supporting figure 21. FTIR of evolved gases during heating of NaTIM at 550 (blue), 710 (purple) and 750 (red) °C.

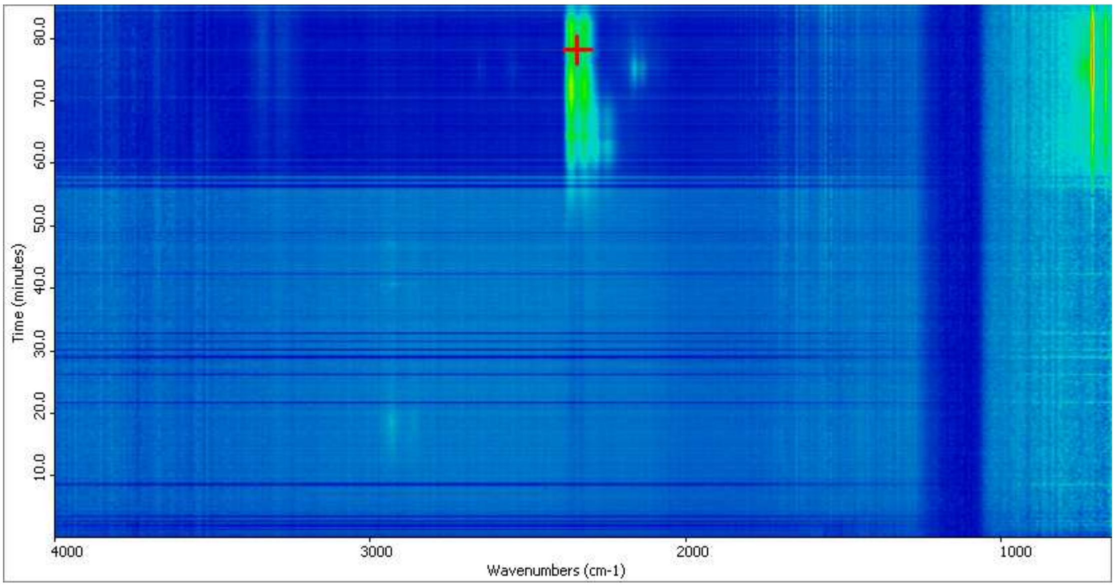

Supporting figure 22. FTIR spectra of evolved gases during TG of NaTCP. The legend below the figure corresponds to peak intensity; where blue to red shift corresponds from negligible to intense peaks.

Supporting table 5. Composition (polymer:salt molar ratio), glass transition temperature (T_g), melting temperature (T_m), for solid-polymer electrolytes undergoing DSC. PEO_{10}NaTIM was not studied due to way different morphology then other presented polymer electrolytes.

**concetration**	**PEO_{10}NaPCPI**	**PEO_{16}NaPCPI**	**PEO_{20}NaPCPI**	**PEO_{50}NaPCPI**
T_g/ °C	-39.8	-36.1	-35.5	-38.0
T_m/ °C	47.1	51.0	50.6	62.1
**concetration**	**PEO_{10}NaTCP**	**PEO_{16}NaTCP**	**PEO_{20}NaTCP**	**PEO_{50}NaTCP**
T_g/ °C	-33.8	-30.7	-30.4	-39.5
T_m/ °C	47.4	52.1	55.9	61.7
**concetration**	**PEO_{10}NaTIM***	**PEO_{16}NaTIM**	**PEO_{20}NaTIM**	**PEO_{50}NaTIM**
T_g/ °C	-	-37.8	-37.7	-44.8
T_m/ °C	-	49.2	54.0	61.0
**concetration**	**$PEO_{10}NaPF_6$**	**PEO_{16} $NaPF_6$**	**PEO_{20} $NaPF_6$**	**PEO_{50} $NaPF_6$**
T_g/ °C	-27.6	-29.4	-39.7	-53.1
T_m/ °C	53.9	57.3	69.9	66.5

a) NaTIM

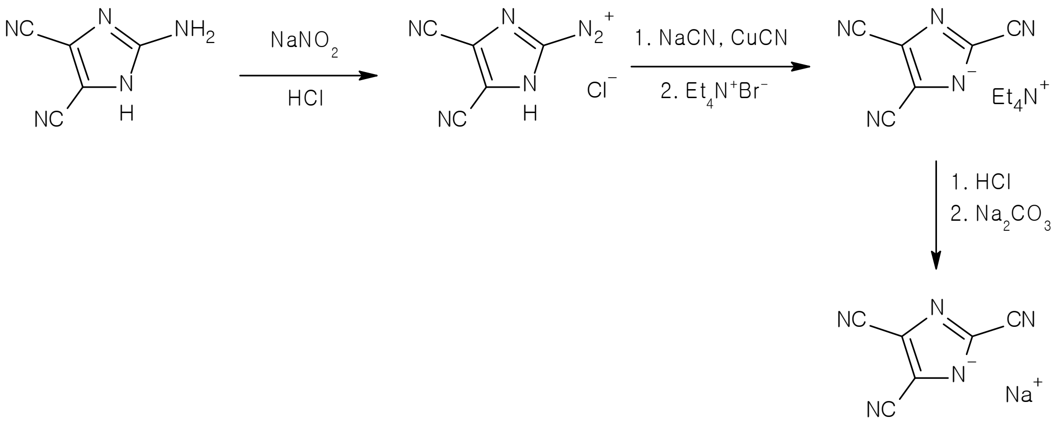

NaTIM

b) NaTCP

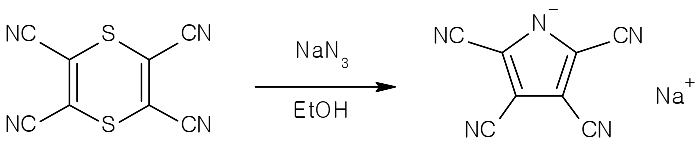

NaTCP

c) NaPCPI

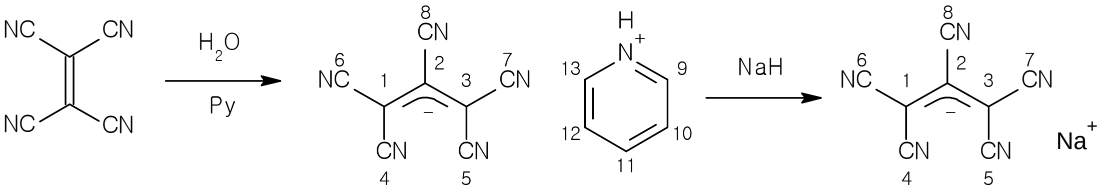

NaPCPI

Supporting figure 23. Synthesis scheme a) NaTIM, b) NaTCP, c) NaPCPI

References

[1] CRYSALIS^{PRO} Software system, Agilent Technologies, Oxford, UK, 2014

[2] Dolomanov, O.V., Bourhis, L.J., Gildea, R.J, Howard, J.A.K. & Puschmann, H. (2009), J. Appl. Cryst. 42, 339-341

[3] Sheldrick, G.M. (2015). Acta Cryst. A71, 3-8

[4] Sheldrick, G.M. (2008). Acta Cryst. A64, 112-122
